# Surveillance in Children and Adolescents with von Hippel-Lindau (VHL)-Related Pheochromocytomas and Paragangliomas: A Survey of MET and Freiburg-VHL Registries in Germany

**DOI:** 10.15586/jkcvhl.v11i4.362

**Published:** 2024-11-20

**Authors:** Fruzsina Kotsis, Marina Kunstreich, Antje Redlich, Kilian Rhein, Athina Ganner, Gerd Walz, Michaela Kuhlen, Elke Neumann-Haefelin

**Affiliations:** 1 Department of Medicine IV – Nephrology and Primary Care, University of Freiburg, Germany;; 2 Department of Pediatrics, Otto-von-Guericke University Magdeburg, Magdeburg, Germany;; 3 Pediatrics and Adolescent Medicine, Faculty of Medicine, University of Augsburg, Augsburg, Germany

**Keywords:** children, paraganglioma, pheochromocytoma, surveillance, VHL

## Abstract

Early identification of patients at risk with von Hippel-Lindau (VHL) syndrome-related pheochromocytoma and paraganglioma (PPGL) is crucial to prevent morbidity. We investigated the current surveillance recommendations in VHL-related PPGL in children and adolescents. German Pediatric Oncology and Hematology–Malignant Endocrine Tumor registry (GPOH-MET) and Freiburg-VHL registry (1996–2022). In all, 75 patients (aged 0–18 years) with VHL syndrome were analyzed and 52 were in the Freiburg screening/surveillance program (median follow-up: 11.5 ± 0.94 years), including annual hormone level measurements, eye examination (starting at the age 6 years), and MRI of the abdomen and central nervous system (CNS) (starting at the age of 12 years). Retrospective analysis of clinical outcomes and descriptive statistics was performed. Of the 75 patients, 60 had a previous clinical diagnosis of PPGL with subsequent genetic testing, and 63% had a positive family history. In spite of having positive family history, large variations of timings between genetic and clinical diagnosis (range: -9 to +40 years) were observed. The mean age of first PPGL was 12.4 ± 0.41 years (range: 4–18 years). Recurrence of PPGL was common (46%; range: 2–7 per patient), and that of other tumors occurred: hemangioblastomas (73%), retinal angiomas (58%), renal cell carcinomas (21%), and pancreatic neuroendocrine tumors (12%). VHL-related PPGL appeared by the age of 12 and recurrences were observed frequently. Hemangioblastomas and retinal angiomas were common. In spite of a positive family history, VHL diagnoses were delayed. Because of high tumor proportions of affected families with children, it needs an optimization of the surveillance framework to enhance compliance and minimize anxiety and worse disease outcomes.

## Introduction

Von Hippel-Lindau disease (VHL; OMIM #193300) is a multi-systemic tumor predisposition syndrome characterized by the occurrence of benign and malignant neoplasms. In addition to retinal angioma (RA), and cerebellar and spinal hemangioblastomas (HBL), the most common tumors are pheochromocytoma (PHEO), paraganglioma (PGL), and renal cell carcinoma (RCC), but pancreatic neuroendocrine tumors (pNETs), endolymphatic sac (petrous bone) tumors (ELSTs), cystadenomas of the epididymis, and cysts of the pancreas and kidneys also occur (1). The incidence of VHL is estimated at 1:36,000 with a lifetime penetrance of approximately 100% at the age of 75 years (2).

Most VHL-associated tumors manifest in the third and fourth decades of life. Children and adolescents, however, are particularly vulnerable, as pheochromocytoma and paraganglioma (PPGL) and HBL remain clinically hidden for a long time and only manifest with the onset of severe clinical manifestations. Furthermore, malignancies occurring in childhood and adolescence are more potential to have an unfavorable course (3, 4). Although most tumors are histologically classified as benign, they may be associated with significant morbidity because of mass effects (loss of visual acuity in RA) and (neuro) surgical interventions (5, 6). The mortality of VHL patients is essentially determined by the occurrence of RCC, pNET, and cerebellar HBL. However, mortality is strongly reduced with the introduction of multidisciplinary care ensuring early diagnosis and consistent surveillance strategies (7–9). Early identification and genetic screening of at-risk individuals are therefore considered key to preventing morbidity and reducing mortality. Families with known VHL variants are strongly recommended for the early examination of children and, if necessary, to start regular screening for disease manifestations at the age of 5 years.

Surveillance for VHL should be lifelong; existing recommendations from various groups primarily focus on adulthood (10–12). In 2017, a multi-professional expert group published recommendations specifically for children and adolescents (13). Screening is performed at 1- or 2-year intervals based on the current stage of tumor and the risk of further progression. Screening is based on the tumor spectrum expected in childhood and adolescence, the earliest age of manifestation, the risk, the assumed growth rate, and the potential consequences of tumor progression (14). The new VHL guidelines of 2023 emphasize the importance of early surveillance measures for patients with positive family history (15). Of note, the knowledge of a lifelong increased tumor risk and the surveillance examinations pose emotional and logistic challenges to affected families, requiring a critical risk-benefit assessment.

Substantial experience in surveillance and treatment over the last 30 years in Freiburg, Germany, has resulted in the “Freiburg screening protocol” for VHL patients. Children and adolescents diagnosed with PPGL are registered in the German Pediatric Oncology and Hematology–Malignant Endocrine Tumor registry (GPOH-MET) (4). In a joint analysis of data from the GPOH-MET and VHL registries, we investigated the current tumor surveillance in children and adolescents with VHL syndrome. We inquired whether surveillance examinations and intervals allow an early diagnosis in childhood, including genotype–phenotype correlations and whether these screenings reduce morbidity in childhood and adolescence.

## Methods

### Patients

The GPOH-MET cohort includes all children and adolescents aged 0–18 years with histologically confirmed PPGL and genetically confirmed VHL syndrome reported to the GPOH-MET study center since 1997 until December 31, 2021. Details of the GPOH-MET 97 study protocol, the GPOH-MET registry, and data collection are provided elsewhere (4). The GPOH-MET 97 and GPOH-MET 2013 databases were approved by the ethics committees of the University of Luebeck, Germany (approval No. 97125) and Otto-von-Guericke University, Magdeburg, Germany (approval No. 174/12). Written informed consent was obtained from patients aged 15 years or older and/or their parents or legal guardians, as appropriate. This study was approved by the responsible ethics committee of the Ludwig Maximilian University of Munich, Germany (approval No. 21-0963).

The Freiburg-VHL registry (since 1992) cohort included patients with genetically confirmed VHL and at least one outpatient visit to the Freiburg VHL center. Written informed consent was obtained from all patients in the VHL registry. The study was approved by the responsible ethics committee of the University of Freiburg, Germany (approval No. 79/20). Data of 500 adults and 153 children/adolescents (aged 0–18 years) were recorded in the Freiburg-VHL registry (data freeze March 28, 2022). Furthermore, 10 patients contacted the Freiburg VHL center only for genetic testing without follow-up. These patients were also included in the analysis and the clinical data were merged with the GPOH-MET registry.

### Clinical data and surveillance protocol

Demographic and clinical information was obtained from the registry and medical records at baseline and during follow-up. According to the current international recommendations, the Freiburg screening protocol of surveillance includes annual hormone level measurements (plasma and/or 24-h urine metanephrines), eye examination (starting at the age of 6), imaging of the abdomen and central nervous system (CNS) (starting at the age 12) usually by magnetic resonance imaging (MRI) and positron emission tomography (PET) scanning with ^68^Ga-peptides. PPGL was diagnosed by histology, when available, or through the combination of biochemical assays and MRI imaging. The clinical follow-up data collected in the Freiburg-VHL registry were available for 52 patients. Patients had at least two screenings at the VHL center, with on average of 1.2 screening annually. Of the 21 overlap patients of the two registries, only baseline data and no follow-up data were available for 10 Freiburg-VHL and 13 GPOH-MET patients.

### Statistical analyses

The acquired data of the two registries were merged and analyzed in Excel. According to the ethical vote for the calculation of descriptive statistics, patients’ anonymized data were used after merging of datasets. Results were presented as mean ± standard deviation, except where otherwise mentioned.

## Results

### Patient characteristics and genetics at baseline

**Figure 1:**
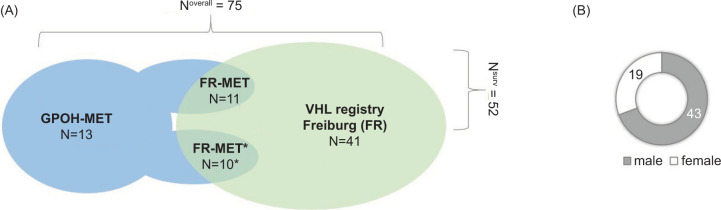
Study population overview: VHL-related PPGL in childhood. (A) Summary of patients in the German Pediatric Oncology and Hematology–Malignant Endocrine Tumor registry in Magdeburg (GPOH-MET) and Freiburg-VHL registry between 1996 and 2022. Inclusion criteria: at least one PPGL manifestation with age <18 years, genetic confirmation of VHL. Overlap of patients, n = 11. Both registries contain clinical and genetic data. Overlap of patients, n = 10. ^*^Genetic data from the Freiburg-VHL registry and clinical data from the GPOH-MET registry without follow-up data. (B) Sex distribution of patients. PPGL: pheochromocytoma and paraganglioma.

**Figure 2:**
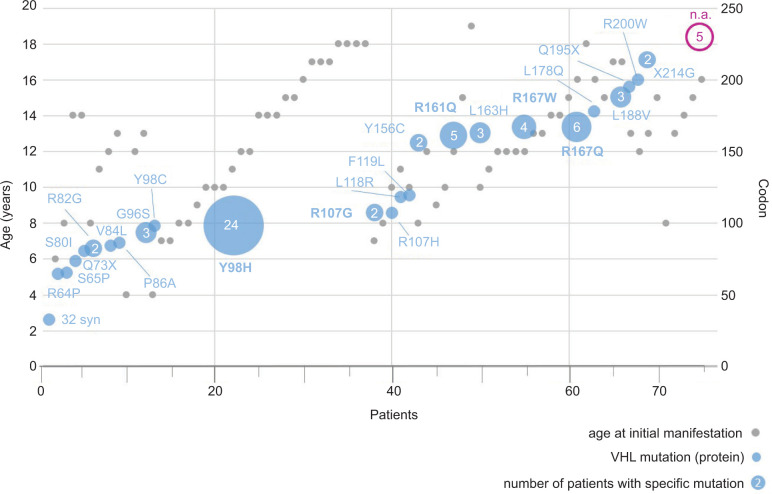
VHL mutations of patients with PPGL in childhood. Notes. Numbers on the X-axis: individual patients. Gray circles and Y-axis on the left: age at first PPGL manifestation. Y-axis on the right: codon numbers of VHL mutation. VHL mutations of the protein level are presented. The size of blue circles represents patient numbers with the corresponding mutation, and if >2, then the number is included. Genetic diagnosis was not available (n.a.) for five patients, but with proven VHL mutation and clinical manifestation in the family, they were not excluded from the analysis. PPGL: pheochromocytoma and paraganglioma.

**Figure S1:**
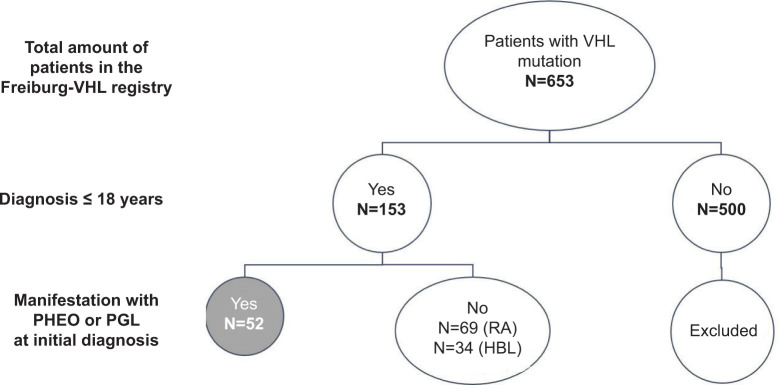
Overview of patients in the VHL-Freiburg registry.

**Figure S2:**
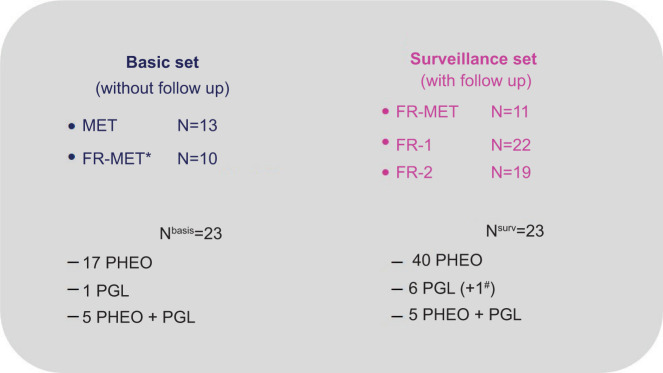
Patients with initial PPGL manifestation are included in the study. Patient numbers and PPGL manifestations are shown. Surveillance set FR-1: First visit and PPGL manifestation of patients in Freiburg as a child. Surveillance set FR-2: first visit of patients in Freiburg as adults with reported childhood PPGL. Out of the overlap of 21 patient in both registries, for 11 patients follow-up data (surveillance set: FR-MET) and full screening for other manifestations were available; for 10 patients, only baseline screening was available (basis set: FR-MET^*^). For 13 patients in the GPOH-MET (MET), only basic screening was available. ^#^Histologically not confirmed at the end of the study. PHEO: pheochromocytoma; PGL: paraganglioma; PPGL: pheochromocytoma and paraganglioma; GPOH-MET (MET): German Pediatric Oncology and Hematology–Malignant Endocrine Tumor registry in Magdeburg; FR: Freiburg-VHL registry.

We identified 75 patients (57% females) with genetically confirmed VHL and PPGL diagnosed before 18 years in both registries. Of these, 41 patients were detected only in the Freiburg-VHL registry, 13 in the GPOH-MET registry, and 21 patients in both registries (Figure 1). GPOH-MET registry patients were usually manifested with PPGL and afterwards diagnosed with VHL. Patients of the Freiburg-VHL registry (Figure S1) either initiated surveillance before the age of 18 years (FR-1, n = 22) or the initial PPGL/VHL was diagnosed elsewhere, but patients contacted the center for genetic testing/surveillance after the age of 18 years (FR-2, n = 19) and had reported having PPGL in childhood. Of the overlap of 21 patient in both registries, follow-up data (FR-MET) and full screening for other manifestations were available for 11 patients, and only baseline screening (FR-MET^*^) was available for 10 patients. Clinical screening at baseline (basis set: MET and FR-MET^*^) was available for 23 patients and surveillance with follow-up data (surveillance set: FR-1, FR-2, and FR-MET) was available for 52 patients. As shown in Supplementary Figure S2, most of the patients were diagnosed with PHEO only (57; 76%) or with PHEO and PGL (10; 13%); PGL alone was less common (7; 9%). As shown in Supplementary Table S1, we observed only missense mutations (except one silent mutation *c.93G>A*) with the most common genotype (24; 32%) of *c.292T>C* corresponding to *p.Tyr98His* (*p.Y98H*, designated as “Black Forest” founder mutation). The other missense mutations affected a large range of codons (32–214) in single patients. Further mutation hotspots affected codons 161 and 167 (*c.R161Q*, n = 5; *c.R167W*, n = 6; or *c.R167Q*, n = 4). We observed a broad age distribution of first PPGL manifestation across different mutations (Figure 2). For example, in the most common mutation *c.292T>C* (*p.Y98H*), the age range of the first manifestation was 6–18 years. Although genetic diagnosis was not available for five patients, they were not excluded from the analysis because of the proven VHL mutation and clinical diagnosis in the family.

### Clinical characterization

**Figure 3:**
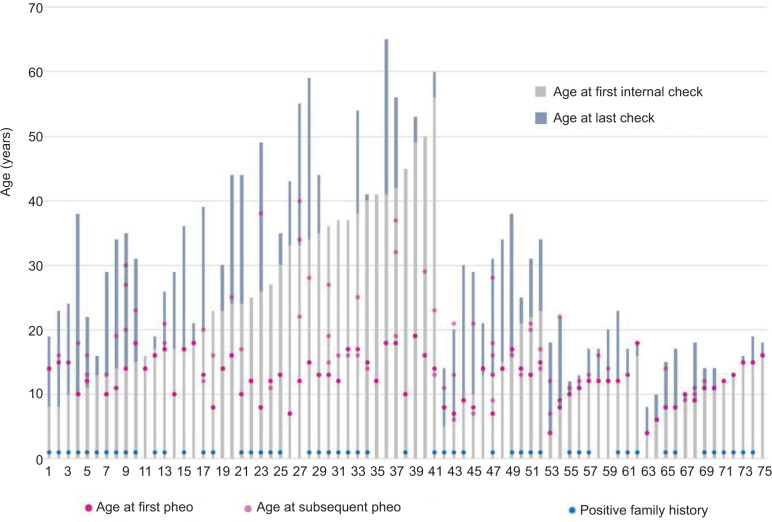
PPGL manifestation at baseline and during surveillance screen. Notes. Ages of patients are plotted at baseline (gray bar “first check”) and during surveillance (blue bar “difference of age at first and last check”). Circles show age of initial PPGL manifestation (pink circles) and recurrence of PPGL (“subsequent pheo”: light pink circles). Numbers on the X-axis: individual patients. Patient number 1-41, surveillance set FR-1: first visit and PPGL manifestation of patients in Freiburg as a child. Surveillance set FR-2: first visit of patients in Freiburg as adults with reported PPGL in childhood. Out of the overlap of 21 patient in both registries, for 11 patients, follow-up data (patient number 42-52, surveillance set: FR-MET) and full screening for other manifestations were available. For 10 patients, only baseline screening was available (patient number 53-62, basis set: FR-MET*). For 13 patients in the GPOH-MET (patient number 63-75, MET), only basic screening was available. PHEO: pheochromocytoma; PGL: paraganglioma; PPGL: pheochromocytoma and paraganglioma.

The overview of PPGL manifestations with age and source (anamnestic or during surveillance) of different registries (Freiburg or GPOH-MET) is presented in Figure 3. Of the 75 patients, 37 (49%) were diagnosed with PPGL prior to the age of 12 years. Recurrent tumors were reported (age range 7–28 years): once in 35 (47%) patients (age range: 4–38 years) and twice in 14 (19%) patients (age range: 7–34 years); maximum number of recurrent tumors were seven in one patient (see patient No. 47, five plus two operations at the age of 13 years). In our analysis, this patient was the only patient with documented metastasis (lymph nodes) at the age of 7 years, and had received chemotherapy and irradiation. In spite of positive family history in the majority of patients, genetic testing was rarely the reason for contacting the VHL-specialized medical care unit. Two patients with PPGL were diagnosed prior to the age of 6 years. Both had a negative family history and were symptomatic (seizure or sweating, headache, stomachache, and weakness).

### Characteristics based on method of detection

**Figure 4:**
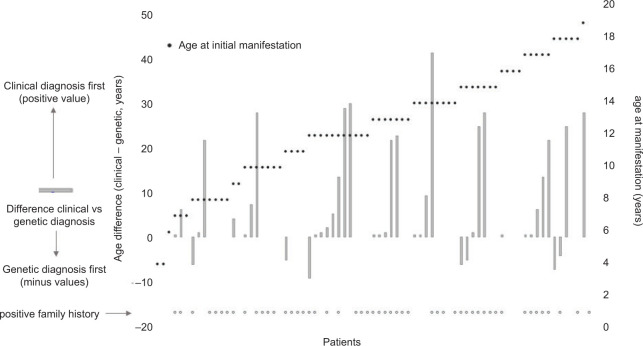
Timing of the genetic and clinical diagnosis. Clinical diagnosis includes first PPGL manifestation and surgery. Bar plots: time difference between clinical and genetic diagnoses (left Y-axis). Negative results represent the time course: first genetic diagnosis followed by clinical manifestation. Positive results represent the time course: first clinical manifestation followed by genetic diagnosis. For 32 patients, no data on the year of genetic testing were available.

The timing of genetic and clinical diagnosis varied widely between patients. Age of manifestation, family history, and difference between clinical versus genetic diagnosis are plotted in Figure 4. Irrespective of the age of manifestation and positive family history, most of the patients were first diagnosed clinically (positive values in Figure 4) and genetic diagnosis was executed later (age range 0.5–40 years). Patients first diagnosed genetically had disease manifestation at the age of 0–9 years (negative values in Figure 4). Overall, 60 patients (80%) contacted the VHL-specialized medical care unit because of clinical manifestations (symptoms, positive MRI findings, and operation), and 38 patients (63%) had positive family history. In 12 patients (16%) only, initial diagnosis of VHL syndrome was performed genetically, mostly in the FR-1 and FR-MET sets (Table 1).

**Table 1:** Overview of the initial diagnostic stratified by family history.

Data set	Family history	Initial diagnostic					
		Clinic		Genetic		Overall	
		n	%	n	%	n	%
FR-1+2 (n=41)	overall	32	78.0	9	22.0	41	100.0
	pos	21	51.2	8	19.5	29	70.7
	neg or n.a	11	26.8	1	2.4	12	29.3
FR-MET (n=21)	overall	18*	85.7	3	14.3	21	100.0
	pos	11	52.4	3	14.3	14	66.7
	neg or n.a	7	33.3			7	33.3
MET (n=13)	overall	13	100.0			13	100.0
	pos	8	61.5			8	61 .5
	neg or n.a	5	38.5			5	38.5
overall (n=75)	overall	63*	84.0	12	16.0	75	100.0
	pos	40	53.3	11	14.7	51	68.0
	neg or n.a	23	30.7	1	1.3	24	32.0

Surveillance set FR-1: first visit and PPGL manifestation of patients in Freiburg as a child. Surveillance set FR-2: first visit of patients in Freiburg as adults with reported PPGL in childhood. Out of the overlap of 21 patient in both registries, for 11 patients, follow-up data (surveillance set: FR-MET) and full screening for other manifestations were available, but for 10 patients, only baseline screening was available (basis set: FR-MET^*^). For 13 patients in the GPOH-MET (MET), only basic screening was available.

* For three patients, no data were available for the reason of initial diagnostics.

**Figure S3:**
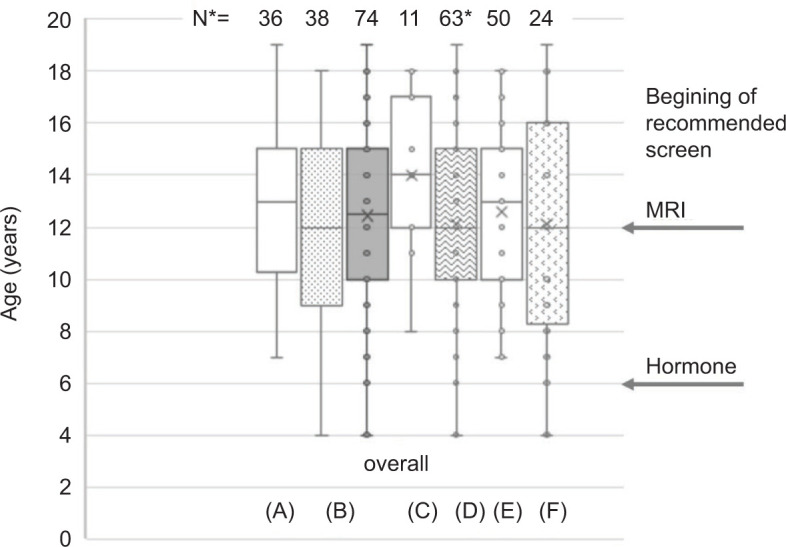
Overview of the age at first PPGL manifestation. Barplots show age at the first PPGL manifestation; N: number of patients with first PPGL manifestation. Overall (dark barplot in the middle), data of 74 patients are included. ^*^Missing = 1 patient (diagnosed clinically with PGL, histologically not confirmed at the end of the study). Subgroups (mean ± SD): (A) First manifestation under surveillance (12.0 ± 3.8 years); (B) first manifestation before baseline screening (12.9 ± 3.2 years); (C) reason of diagnosis, genetic (14.0 ± 3.1 years); (D) reason of diagnosis, clinical (12.1 ± 3.6 years), ^**^for three patients no data for initial diagnosis were available; (E) family history positive (12.6 ± 3.0 years); and (F) family history negative (12.1 ± 4.5 years). Recommended screening with hormone measurements starts at the age of 6 years, and MRI screening starts at the age of 12 years.

The mean age of the first PPGL manifestation was 12.4 ± 0.41 years (range 4–18 years) (Supplementary Figure S3). Initial manifestation age did not differ between patients under surveillance (12.0 ± 3.8 years), compared to manifestation age before initial checkup (12.9 ± 3.2 years). Similarly, no difference was observed regarding patient’s family history (positive vs. negative: 12.6 ± 3.0 years vs. 12.1 ± 4.5 years) or initial manifestation (genetic vs. clinical: 14.0 ± 3.1 years vs. 12.1 ± 3.6 years). Of note, the recommended screening with MRI started at the age of 12 years.

### Follow-up manifestations

**Figure S4:**
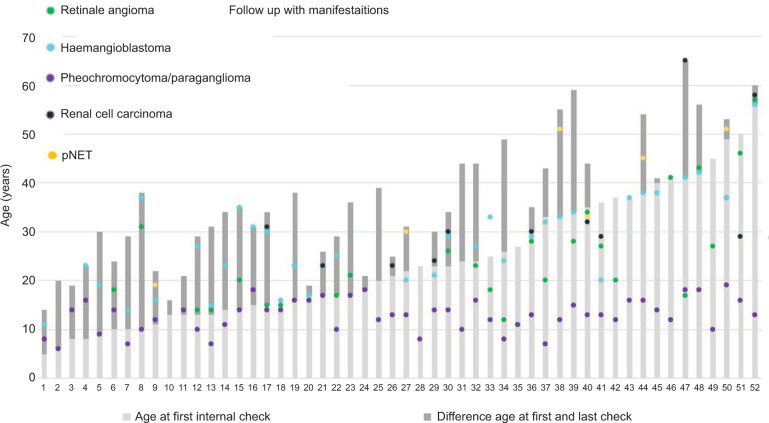
VHL manifestations at baseline and during surveillance. Ages of patients are plotted at baseline (light gray bar “first check”) and during surveillance (dark gray bar “difference in age at first and last check”). Numbers on the X-axis: individual patients. Circles show age at different VHL manifestations. Patient no. 33 had one manifestation (outside of surveillance) after the first screening at the VHL-Freiburg center (diagnosis: hemangioblastoma outside the surveillance). pNET: pancreatic neuroendocrine tumor.

All manifestations at baseline (n = 23) and at the end of follow-up (n = 52) are listed in Table 2. Patients under surveillance had a median observation time of 11.5 years (±0.94) for follow-up. Overall, the most common manifestation was HBL (38; 73%), followed by RA (30; 58%), RCC (11; 21%), and pNET (6; 12%). Supplementary Figure S4 depicts individual patients with VHL manifestations over time. Prior to the age of 18 years, manifestations other than PPGL included RA (11; 21%) and HBL (6; 12%). After the age of 18 years, manifestations other than PPGL included HBL (32; 62%), RA (19; 37%), RCC (11; 21%), and pNET (6; 12%).

**Table 2:** Overview of all VHL manifestations

	Surveillance set (N=52)			Basis set (n=23)		operations (min. 1)		Overall n (%)
	FR-1 (n=22)	FR-2 (n=19)	FR-MET (n=11/21)	FR-MET* (n=10/21)	MET (n=13)	FR-1	FR-2	75
PPGL	22	19	21*		13	20	19	75/75 (100)
CNS HBL	11	18	9				11	38/52 (73)
RA	9	16	5			11	17	30/52 (58)
RCC	1	7	3				5	11/52 (21)
pNET	1	4	1			1	4	6/52 (12)

All manifestations at the end of follow-up are presented.n: number of patients. Surveillance set FR-1: first visit and PPGL manifestation of patients in Freiburg as a child. Surveillance set FR-2: first visit of patients in Freiburg as adults with reported PPGL in childhood. Out of the overlap of 21 patient in both registries, for 11 patients follow-up data (surveillance set: FR-MET) and full screening for other manifestations were available. For 10 patients, only baseline screening was available (basis set: FR-MET^*^). For 13 patients in the GPOH-MET (MET), only basic screening was available.

* One patient diagnosed with PGL after surgery was not confirmed.

PPGL: pheochromocytoma and paraganglioma; RCC: renal cell carcinoma; pNETs: pancreatic neuroendocrine tumors; RA: retinal hemangioblastomas; CNS: central nervous system, including cerebellar and spinal hemangioblastomas (HBL).

## Discussion

In this study, we analyzed a large cohort of 75 pediatric patients in Germany with VHL-related PPGL, and investigated its natural disease course during the current surveillance recommendations. VHL *-*related PPGL was diagnosed at a median age of 12 years, and recurrent tumors were common. Other manifestations included HBL and RA, which occurred frequently. In spite of a positive family history, VHL diagnoses were delayed as genetic testing was initiated after the onset of clinical manifestations. These results underscored the need for a systematic approach for affected families with children and a close follow-up of patients with VHL disease.

*VHL* mutations commonly lead to PHEOs (50% bilateral) and less frequently to PGLs (13). Consistently, just over 75% of the patients in our cohort had PHEO only. VHL disease has a penetrance of approximately 100% at the age of 75 years (16), and in literature, the frequency of PPGL in these patients is estimated as approximately 10–25% In the Freiburg-VHL registry, we observed a higher occurrence of PPGL in pediatric patients (34%; 52 out of 153 patients), probably due to a referral bias toward the center. Recurrence of PPGL in our analysis was high, with almost 50%. Furthermore, increased metastatic risk in patients with VHL mutations is known (13, 19). This underlines the importance of regular screening and organized surveillance care of patients at risk.

One of our main findings was the large discrepancy (range: –9 to +40 years) between genetic testing and clinical manifestations in spite of a positive family history. Generally, the final diagnosis took a very long time. This was explained by rarity of the disease, which required early referral to specialized centers. Finally, only the genetic diagnosis enables the patients to contact specialized medical care with organized follow-up for VHL. Overall, there is a long path from diagnosis to surveillance with an increased risk of disease progression. Therefore, a genetic-driven, coherent, and individualized patient management plan is the future direction of patient care with VHL *-*related PPGL. The current international VHL guidelines (15), in line with our findings, suggest genetic testing shortly after birth in affected families as well as surveillance of high-risk individuals (with VHL missense variants).

Given the ongoing risks of tumor development and malignant growth with increasing age, longitudinal surveillance is crucial for preventing morbidity and mortality in VHL disease (7, 8). The Endocrine Society, Washington, DC, suggests a personalized management with evaluation and treatment by multidisciplinary teams with appropriate expertise to ensure favorable outcomes (20). Surveillance guidelines differ in the recommended age for the start of screening after birth, or at 2 years or 5 years of age (10, 13, 21). In Freiburg, the surveillance with hormone measurements starts at the age of 6 years, retinoscopy after dilatation at 6 years of age, and MRI screening at the age of 12 years (14). Our data support this approach, as the mean age of PPGL onset was 12.5 years. Recently, new VHL guidelines of 2023 (15) suggest that clinical screening should begin at the age of 2 years (vital clinical manifestations), and the annual biochemical screening must begin at the age of 5 years, especially in children with VHL missense variants (evidence level III). In our cohort, two children aged <6 years presented with PPGL.

During a follow up of 10 years, we observed other VHL manifestations. In 73% patients, HBL occurred with a high frequency, as reported in other studies (60–80%), and the age distribution was similar between children and adults (14, 22). RA were reported in as many as 60% of patients, which was in line with our data (52%) (23, 24). In our study, RCC and pNET occurred with a lower frequency (21% and 12%, respectively), compared to other studies, and only in adults (25).

Germline VHL missense mutations are associated with a high risk of PHEO whereas large deletions or nonsense mutations are rarely observed in patients with PHEO (26, 27). In fact, we observed (except the silent mutation *c.93G>A*) missense mutations only in exon 1 (53%) with the hotspot *c.292T>C* (*p.Y98H*) (28) and in exon 2 (37%) with the hotspots *c.482G>A, 499C>T*, and *500G>A*. These hotspots were previously reported as well with high penetrance (29, 30). Other pathogenic mutations were *c.191G>C, 193T>C, 256C>G*, and *240T>G*.

Our analysis had a special feature of combining the data of two registries with different reference areas, recruitments, and follow-up screening. In the GPOH-MET registry cohort, pediatric patients with PPGL in Germany are registered, while the focus of the Freiburg-VHL registry is specialized care for patient with VHL disease. Interestingly, only a fraction of patients occurred in both cohorts (28%). These results reflect the interdisciplinary and organizational challenges of this multisystem tumor disease. Since regulations involve reporting to the GPOH-MET registry, the recruitment of patients for the Freiburg-VHL registry is proactive (patients personally contact the center for diagnostic or annual examination). One explanation for this distribution could be that patients are more likely to refer to a specialized center (with established management for diagnostic procedure on 1 day) where adequate therapies (specialist disciplines with corresponding expertise in one hospital) are available. Physicians treating patients inform the affected families about the recommendations for regular screening of children, but there is no active reminder or feedback about upcoming examinations for at-risk patients. Another hurdle is that later pediatric patients would have to manage their own screening, which depends on the flow of information within the family.

### Strength and limitations

Our study has several strengths. First, it provides important information on the natural course of VHL *-*related PPGL progression and other manifestations in a large number of pediatric patients under the current surveillance. Second, a high number of patients from two registries were combined, encompassing a broad reference area in Germany. Third, surveillance data were available for individuals aged <18 years as well as >18 years, allowing subgroup analyses of these groups. Finally, patients were enrolled in a standardized surveillance with annual follow-up visits at a VHL-specialized medical center.

Our study also has potential limitations. First, different referral strategies of the two registries could have biased the analysis. Second, data on follow-up visits were only available for the Freiburg-VHL registry and length of the follow-up period varied between patients. Third, it could also reflect the predominance of the *c.292T>C* (*p.Y98H*) mutation, with a founder effect, as the geographical region of the Freiburg-VHL registry is in the Black Forest. However, this mutation is documented in numerous European and American families as well (28, 30). On the other hand, the fact that a large proportion of individuals having the same founder mutation could compromise the generalizability of these results. Fourth, the retrospective nature of this study lessened the availability of some data. Data on hormone levels and clinical manifestations (e.g., hypertension) were not consistently available in the study population, and this could be addressed in further investigations. Finally, we had no results of other follow-up examinations outside the VHL center.

## Conclusions

VHL-related PPGL occurred at the age 12 years and recurrences were common in 50% cases. In spite of high proportion of positive family history of these patients, the diagnosis relied more on clinical manifestations, with genetic testing conducted later on. The risk of developing PPGLs in VHL-affected family members increases once a family history of PPGL is reported. A framework for patient management is necessary with the education of affected families, active surveillance of manifestations according to international guidelines, and the referral of affected children to VHL-specialized centers. The possibility to follow outcomes via national networks and/or follow-up studies may enable optimization of the surveillance scheme, thus enhancing compliance and minimizing anxiety and medical complications.

## Figures and Tables

**Table S1:** VHL mutations in the study population.

Patient	VHL mutation (cDNA)	VHL mutation (protein)	Codon
1	93 G>A	32 silence	32
2	191 G>C	R64P	64
3	193 T>C	S65P	65
4	217 C>T	073X	73
5	239 G>T	S80I	80
6,7	244 C>G	R82G	82
8	250 G>C	V84L	84
9	256 C>G	P86A	86
10, 11, 12	277 G>A	G93S	93
13–37	293 A>G	Y98C	98
38	320 G>A	R107H	107
39, 40	319 C>G	R107G	107
41	353 T>G	L118R	118
42	357 C>G	F119L	119
43, 44	467 A>G	Y156C	156
45–49	482 G>A	R161Q	161
50–52	488 T>A	L163H	163
53–56	499 C>T	R167W	167
57–62	500 G>A	R167Q	167
63	533 T>A	L178Q	178
64–66	562 C>G	L188V	188
67	583 C>T	Q195X	195
68	598 C>T	R200W	200
69–70	640 T>G	X214G	214
71–75	NA	NA	NA

Molecular data on cDNA and protein level of the study population.

## Data Availability

Public posting of individual-level participant data is not covered by patient’s informed consent form. Upon approval of a scientific project proposal, collaborating scientists may receive a dataset containing pseudonyms, as indicated in patient’s informed consent form and as approved by ethics committees.

## Author Declaration

Authors had nothing to disclose.
